# Functional Evaluation of Pomegranate (*Punica granatum*) Juice Byproducts as Dietary Additives in Red Seabream (*Pagrus major*): Effects on Growth Performance, Antioxidant Response, Immunity, and Resistance to *Edwardsiella tarda*

**DOI:** 10.3390/antiox15040517

**Published:** 2026-04-21

**Authors:** Ki-Tae Kim, Tae Hoon Lee, Hwa Yong Oh, Da Ye Kang, Do Hyun Kwon, Young Wook Kim, Bo Seong Gu, Dona Thilini Udarika Samaraweera, Hee Sung Kim

**Affiliations:** 1 Southeast Sea Fisheries Research Institute, National Institute of Fisheries Science, Tongyeong 53017, Republic of Korea; oysterkim@korea.kr; 2 Department of Marine Biology and Aquaculture, Gyeongsang National University, Tongyeong 53064, Republic of Korea; xogns2357@gnu.ac.kr (T.H.L.); oho1203@gnu.ac.kr (H.Y.O.); ekdp3292@gnu.ac.kr (D.Y.K.); wngsod@gnu.ac.kr (D.H.K.); kimmangkkong@gnu.ac.kr (Y.W.K.); gbs9131@naver.com (B.S.G.); dthilini98@gmail.com (D.T.U.S.)

**Keywords:** pomegranate byproduct, *Pagrus major*, antioxidant response, immune function, *Edwardsiella tarda*

## Abstract

This study evaluated the potential of pomegranate (*Punica granatum*) juice byproducts (PJB) as a functional dietary additive for juvenile red seabream (*Pagrus major*). Four experimental diets were formulated to contain various levels of PJB (0, 2.5, 5.0, and 10.0 g/kg) and fed to fish with an initial body weight of 7.0 ± 0.01 g for 8 weeks. Growth performance, feed utilization, digestive enzyme activity, whole-body composition, plasma biochemical parameters, antioxidant responses, immune parameters, and resistance to *Edwardsiella tarda* infection were evaluated. Fish fed the diet containing 2.5 g/kg PJB exhibited significantly higher final body weight, weight gain, and specific growth rate compared with the control group and those with higher PJB doses, whereas feed intake, feed efficiency, and protein efficiency ratio were not significantly affected by dietary treatment. Intestinal trypsin and lipase activities were significantly elevated in the PJB2.5 group, whereas amylase activity remained unchanged. Whole-body proximate composition and plasma biochemical parameters, including aspartate aminotransferase, alanine aminotransferase, total cholesterol, glucose, and total protein, were not significantly influenced by dietary PJB supplementation. Dietary inclusion of PJB at 2.5 g/kg also significantly enhanced plasma antioxidant enzyme activities, as evidenced by increased superoxide dismutase and glutathione levels, while catalase activity was elevated in fish fed the PJB2.5 and PJB5 diets. Innate immune responses were also stimulated, with significantly higher serum lysozyme activity and interleukin-1 levels observed in fish fed the PJB2.5 diet. Following experimental challenge with *E. tarda*, fish fed diets containing 2.5 and 5.0 g/kg PJB exhibited significantly higher cumulative survival than the control group. In conclusion, dietary supplementation with PJB at 2.5 g/kg improved growth performance, digestive capacity, antioxidant status, innate immune responses, and disease resistance in juvenile *P. major* without adverse physiological effects.

## 1. Introduction

Intensive aquaculture practices have expanded rapidly, which has consequently increased the susceptibility of farmed fish to environmental stress and infectious diseases, particularly during early developmental stages. The high stocking densities, fluctuating water quality, and frequent handling associated with aquaculture can compromise immune function and physiological stability, leading to reduced growth performance and increased disease outbreaks [1,2,3,4]. There is thus growing interest in dietary strategies that enhance the growth and health of farmed fish while reducing the reliance on antibiotics and synthetic chemotherapeutics [5,6]. In this context, functional feed additives derived from natural sources have attracted increasing attention as sustainable alternatives for improving fish performance and disease resistance. Among these, agroindustrial byproducts represent a promising resource, as they contribute to circular bioeconomy initiatives while providing bioactive compounds with antioxidant and immunomodulatory properties [7,8,9]. The valorization of such byproducts reduces environmental waste and offers cost-effective ingredients for aquafeed formulations.

Pomegranate (*Punica granatum*) juice byproducts (PJB), consisting primarily of peel and seeds discarded during juice processing, account for nearly half of the total fruit biomass and are often treated as waste. However, these byproducts contain considerable amounts of bioactive compounds, including polyphenolic compounds, flavonoids, ellagitannins, and vitamin C, which are associated with strong antioxidant, antimicrobial, and immunomodulatory activities [10,11,12]. Notably, many of these functional properties are retained even without solvent extraction, suggesting that PJB may be suitable for direct inclusion in animal feed. Indeed, previous studies have demonstrated the beneficial effects of pomegranate-derived products on terrestrial livestock and various aquaculture species, including improvements in growth performance, antioxidant status, and immune responses [13,14,15]. Nevertheless, most studies performed to date have focused on freshwater species or have utilized pomegranate peel extracts rather than whole juice byproducts. Moreover, there is very little information on the application of PJB to the diets of marine finfish.

Red seabream (*Pagrus major*) is one of the most economically important marine finfish species in East Asia, particularly in Japan, the Republic of Korea, and China. In the Republic of Korea alone, aquaculture production of this species reached approximately 6474 metric tons in 2024, highlighting its substantial economic value [16]. Despite its commercial importance, juvenile *P. major* is highly susceptible to environmental stress and bacterial diseases, especially infections caused by *Edwardsiella tarda*, which can result in significant economic losses in the aquaculture industry [17,18,19]. Improving growth performance and innate immune defense during the juvenile stage is therefore critical for the sustainable production of this species.

Against this background, and given the bioactive potential of PJB and the lack of information regarding their application in marine finfish, the present study was planned with the goal of evaluating the effects of dietary supplementation with different levels of PJB on juvenile *P. major*. Specifically, this study investigated the effects on variables such as growth performance, feed utilization, digestive enzyme activity, whole-body composition, plasma biochemical parameters, antioxidant responses, immune parameters, and resistance to *E. tarda* infection.

## 2. Materials and Methods

### 2.1. Ethics Statement

All experiments were performed following the guidelines of the International Animal Care and Use Committee of Gyeongsang National University, Republic of Korea (approval code: GNU-240325-E0065; date of approval: 25 March 2024).

### 2.2. Preparation of PJB and Phytochemical Characterization

Residual materials left over after commercial extraction of the juice of *P. granatum* were collected from a local juice vendor in Jeju-si, Republic of Korea. Specifically, after comprehensive washing, the fruits were juiced using a commercial juicer (Model H-300L-DBFC03; Hurom Co., Seoul, Republic of Korea), and the resulting byproducts were dried at 20 °C for 48 h using an agricultural dryer (KED-M07D1; Kiturami Co., Seoul, Republic of Korea). The dried material was then pulverized into a fine powder and stored at 4 °C until further processing.

Vitamin C concentration was assessed via high-performance liquid chromatography (HPLC, Agilent 1200 Series; Agilent Technologies, Santa Clara, CA, USA) equipped with a UV detector (254 nm) using a mobile phase of 0.05 M KH_2_PO_4_ buffer (pH 2.8) at 1.0 mL/min. Total phenolic content was quantified using the Folin–Ciocalteu assay [20], whereas total flavonoids were determined through aluminum nitrate colorimetric analysis [21]. Antioxidant properties were evaluated using 2,2′-azinobis-(3-ethylbenzothiazoline-6-sulfonic acid) (ABTS) and 1,1-diphenyl-2-picrylhydrazyl radical scavenging assays. For both of these assays, IC_50_ values were derived by plotting absorbance changes against sample concentrations. Detailed chemical properties are provided in Table 1.

Quantitative determination of phytochemical constituents of the PJB revealed vitamin C content of 10.21 ± 2.240 mg/100 g, total phenolic content of 16.15 ± 4.881 mg GAE/100 g, and total flavonoid concentration of 14.45 ± 6.794 mg QE/g. PJB also exhibited a dose-dependent scavenging effect with an IC_50_ value of 4.8 µg/mL, whereas the ABTS assay showed similar antioxidant efficiency with an IC_50_ of 5.0 µg/mL.

### 2.3. Experimental Diet Formulation

To evaluate the physiological effects of dietary PJB on juvenile *P. major*, four experimental diets containing different levels of PJB were formulated, namely, 0 g/kg (PJB0, control), 2.5 g/kg (PJB2.5), 5.0 g/kg (PJB5), and 10.0 g/kg (PJB10), on a dry matter basis (Table 2). Dry ingredients, including sardine meal, dehulled soybean meal, wheat flour, and the specified levels of PJB, were first weighed individually and subsequently mixed thoroughly to obtain a homogeneous blend. Lipid sources (fish oil and soybean oil) were then incorporated and blended thoroughly into the dry mix. Finally, distilled water was gradually introduced to adjust the dough consistency to a level suitable for forming pellets. The prepared feed dough was pelletized using a chopper fitted with a 3.0 mm die (SL Machinery, Incheon, Republic of Korea). The pellets were then spread evenly and dried in a forced-air dryer at 20 °C for 48 h (Model KED-M07D1; Kiturami Co. Ltd., Seoul, Republic of Korea). Once dried, they were sealed in polyethylene bags and stored at 20 °C until the start of the feeding trial.

### 2.4. Feeding Trial Condition and Design Experiment

Juvenile *P. major* with a mean initial body weight of 7.0 ± 0.01 g were obtained from a commercial hatchery (Tongyeong, Gyeongsangnam-do, Republic of Korea). Fish were acclimated for two weeks in a flow-through seawater system at the Marine Bio-Education and Research Center at Gyeongsang National University (Tongyeong, Gyeongsangnam-do, Republic of Korea), during which time they were fed a commercial diet (Jeil Feed Co., Haman, Gyeongsangnam-do, Republic of Korea). After fasting for 24 h, 420 individuals were randomly and equally allocated into 12 fiberglass tanks (200 L capacity; 35 fish per tank). Each treatment was performed in triplicate. Fish were maintained under constant environmental conditions as follows: water temperature (21.4 ± 0.45 °C), salinity (32.2 ± 0.26 psu), and dissolved oxygen (7.5 ± 0.81 mg/L). Photoperiod was set to 9L:15D using overhead LEDs. Fish were fed to apparent satiation twice daily at 09:00 and 17:00 for 8 weeks. Daily feed consumption (FC) and mortality were monitored, and tanks were cleaned once daily.

### 2.5. Growth and Feed Utilization Indices

Following the feeding period, all fish were fasted for 24 h. Body weights were measured for all fish in each tank. Ten fish per tank were anesthetized with tricaine methanesulfonate (MS-222, 150 ppm) and used for biometric assessments. Growth metrics were calculated using the following standard equations:

Survival rate (SR, %) = (Final fish number/Initial fish number) × 100

Weight gain (WG, g) = Final weight − Initial weight

Specific growth rate (SGR, %/day) = [ln(Final weight) − ln(Initial weight)]/days × 100

FC (g) = Total feed intake/Number of fish

Feed efficiency (FE) = WG/Feed intake

Protein efficiency ratio (PER) = WG/Protein intake

Condition factor (CF) = Weight × 100/Length^3^

Hepatosomatic index (HSI, %) = (Liver weight/Body weight) × 100

Viscerosomatic index (VSI, %) = (Viscera weight/Body weight) × 100

### 2.6. Digestive Enzyme Activity

At the conclusion of the feeding experiment, three fish were randomly selected from each tank (n = 3 per dietary group) and anesthetized using tricaine methanesulfonate (MS-222, 150 ppm; Sigma-Aldrich, St. Louis, MO, USA). These specimens were previously used for biometric assessments, in order to maintain consistency across physiological and biochemical parameters. Following dissection, the abdominal cavity was opened, and the entire intestinal tract was carefully removed. The intestines were gently rinsed with prechilled phosphate-buffered saline (PBS, pH 7.4) and immediately placed on ice to minimize enzymatic degradation. Intestinal tissues were homogenized in a 10-fold volume (*w*/*v*) of ice-cold 0.86% physiological saline using a Tissue Lyser II system (QIAGEN, Venlo, the Netherlands) operated in an ice-cooled environment. The homogenates were centrifuged at 13,000 rpm for 10 min at 4 °C, after which the obtained supernatants were collected for enzyme activity assays. The activities of the digestive enzymes amylase, trypsin, and lipase were determined using commercial enzyme-linked immunosorbent assay kits (MyBioSource Inc., CA, USA), following the manufacturer’s instructions. All enzyme activities were standardized to total protein content and reported as milliunits per milligram of protein (mU/mg protein).

### 2.7. Blood Collection and Sample Preparation

At the end of the 8-week feeding trial, five fish from each tank were randomly selected for blood sampling following a 24 h fasting period. The fish were anesthetized with 100 ppm tricaine methanesulfonate (MS-222; Sigma-Aldrich, St. Louis, MO, USA) before blood collection. Blood was drawn from the caudal vein using two types of syringes: heparinized for plasma extraction and nonheparinized for serum preparation.

For plasma separation, samples collected in heparin-coated syringes were centrifuged at 7000 rpm for 15 min at 4 °C. The resulting plasma was immediately aliquoted and stored at −80 °C for subsequent analyses of general biochemistry and antioxidant enzyme activity. Serum was obtained from blood collected without anticoagulant by allowing clotting at 4 °C for 30 min, followed by centrifugation at 3000× *g* for 5 min. The separated serum was used for immunological assays, including lysozyme activity, immunoglobulin M (IgM), and interleukin-1 (IL-1), and stored at −80 °C until analysis.

#### 2.7.1. Plasma Biochemistry

The collected plasma was used to determine the biochemical parameters. Aspartate aminotransferase (AST), alanine aminotransferase (ALT), total cholesterol (TCHO), total protein (TP), and glucose (GLU) concentrations were measured using an automated clinical chemistry analyzer (Fuji Dri-Chem NX500i; Fujifilm, Tokyo, Japan), following the manufacturer’s guidelines.

#### 2.7.2. Antioxidant Enzyme Activity

To evaluate antioxidant status, plasma levels of superoxide dismutase (SOD), catalase (CAT), and glutathione (GSH) were measured using commercial ELISA kits (MyBioSource Inc., CA, USA). All analyses were conducted in accordance with the manufacturer’s protocols. Absorbance values were read using a MULTISKAN GO microplate reader (Thermo Scientific, Vantaa, Finland). Results are expressed as units per milliliter (U/mL) for SOD, nanomoles per minute per milliliter (nmol/min/mL) for CAT, and micromolar (µM) concentrations for GSH.

#### 2.7.3. Immune Parameters

Lysozyme activity was determined using a previously described turbidimetric method [22]. A volume of 100 µL of serum was mixed with 1.9 mL of *Micrococcus lysodeikticus* suspension (0.2 mg/mL; Sigma, St. Louis, MO, USA) in 0.05 M sodium phosphate buffer (pH 6.2). Absorbance at 530 nm was recorded at 25 °C over a 60 min period using a spectrophotometer (Thermo Fisher Scientific, Tewksbury, MA, USA). One unit of activity was defined as the amount of enzyme reducing absorbance by 0.001 per minute.

Quantification of serum IgM and IL-1 levels was performed using ELISA kits: IgM and IL-1 were measured using kits from MyBioSource Inc. (San Diego, CA, USA). The assays were conducted following the manufacturer’s protocols, and the results are expressed as mg/mL (IgM) and pg/mL (IL-1).

### 2.8. Proximate Body Composition

To assess whole-body nutrient composition, five fish from each treatment group previously sampled for evaluating growth performance were selected to ensure consistency across the datasets. The fish were rinsed with distilled water to remove surface moisture and impurities, and then eviscerated, chopped, and homogenized into a uniform paste for compositional analysis. Standard procedures outlined by the Association of Official Analytical Chemists [23] were employed to determine moisture, crude protein, crude lipid, and ash contents. Moisture was quantified by oven-drying the homogenized samples at 105 °C for 24 h until a constant weight was achieved. Crude protein content was measured via the Kjeldahl method using a KjelROC Analyzer (KD310-A-1015; OPSIS Liquid LINE, Furulund, Sweden), with nitrogen values converted to protein using a factor of 6.25. Crude lipids were extracted through Soxhlet extraction using a Soxtec™ system (ST 243; FOSS, Hillerød, Denmark). Ash content was assessed by combusting the samples in a muffle furnace at 550 °C for 4 h.

### 2.9. Challenge Test

Following the end of the feeding trial, a bacterial challenge test was conducted to assess the resistance of *P. major* to *E. tarda* infection. From each tank, 10 fish were randomly selected and transferred into individual 50 L tanks designated for the challenge phase. The *E. tarda* strain used in the challenge was obtained from the Korean Culture Collection of Aquatic Microorganisms, maintained by the National Institute of Fisheries Science (Busan-si, Republic of Korea). The bacterium was cultured in tryptic soy broth at 28 °C for 24 h, after which it was diluted with sterile PBS to a final concentration of 1.0 × 10^6^ colony-forming units/mL. The challenge dose (1.0 × 10^6^ CFU/mL) was determined based on preliminary observations of mortality progression and clinical signs following infection. Each fish was intraperitoneally injected with 0.1 mL of the bacterial suspension using a sterile syringe. During the 9-day postinjection period, fish were monitored at 6 h intervals for signs of morbidity and mortality. Water temperature and dissolved oxygen concentration were maintained at 22.7 ± 0.13 °C and 6.9 ± 0.11 mg/L, respectively, using a flow-through seawater system. Dead individuals were promptly removed to prevent secondary infections, and no feed was provided during this period to avoid confounding effects. Survival data were recorded daily and used to evaluate disease resistance among dietary treatments.

### 2.10. Statistical Analysis

All percentage data, including survival and other ratio-based indices, were arcsine square root–transformed prior to statistical testing to stabilize variance. Results are expressed as mean ± standard error. Homogeneity of variances among dietary treatments was confirmed using Levene’s test. Differences among treatments were analyzed using one-way ANOVA. When significant differences were detected, Tukey’s honestly significant difference test was applied for pairwise comparisons. The significance threshold was set at *p* < 0.05. For the bacterial challenge trial, cumulative SRs were analyzed using Kaplan–Meier survival curves. The statistical significance of differences among treatments was assessed using both log-rank (Mantel–Cox) and Wilcoxon tests. All statistical analyses were performed using SPSS statistical software (version 27.0; IBM Corp., Armonk, NY, USA).

## 3. Results

### 3.1. Growth Performance and Feed Utilization

The results of this study showed that, after 8 weeks of feeding, the growth performance of juvenile *P. major* was influenced by dietary supplementation with PJB (Table 3). SR remained high across all treatments, ranging from 95.2% to 96.2%, with no significant differences observed (*p* > 0.05). However, fish fed the PJB2.5 diet achieved the highest final body weight, which was significantly greater than those of fish fed the PJB0, PJB5, and PJB10 diets (*p* < 0.05). Similarly, WG and SGR showed significant differences among the dietary groups (*p* < 0.05). Juvenile red seabream receiving the PJB2.5 diet exhibited superior WG and SGR, both of which were significantly higher than those of the PJB5 and PJB10 groups. Feed intake (FI), FE, and PER were not significantly affected by the inclusion of PJB in the diet (*p* > 0.05). In addition, body indices, including CF, VSI, and HSI, did not differ significantly among dietary groups (*p* > 0.05).

### 3.2. Digestive Enzyme Activities

The activities of digestive enzymes (amylase, trypsin, and lipase) in the intestine of juvenile *P. major* after the 8-week feeding trial are presented in Table 4. No significant differences were observed in intestinal amylase activity among the dietary groups (*p* > 0.05). However, the activities of trypsin and lipase were significantly affected by the inclusion of PJB in the diet (*p* < 0.05). Fish fed the PJB2.5 diet exhibited the highest trypsin activity, which was significantly higher than that of the control group. Similarly, lipase activity was markedly enhanced in the PJB2.5 group compared with the level in the control group. Although the PJB5 and PJB10 groups also showed numerically higher values of trypsin and lipase than the control, these differences were not statistically significant (*p* > 0.05).

### 3.3. Whole-Body Composition and Hematological Indicators

The whole-body proximate composition of juvenile *P. major* fed experimental diets containing various levels of PJB for 8 weeks is presented in Table 5. No significant differences in moisture, crude protein, crude lipid, or ash contents were observed among the dietary treatments (*p* > 0.05).

Hematological parameters of juvenile *P. major* fed experimental diets with different levels of PJB for 8 weeks are presented in Table 5. No significant differences (*p* > 0.05) were observed among dietary treatments for AST, ALT, TCHO, GLU, and TP. Plasma AST ranged from 58.0 to 63.0 U/L, while the ALT values remained between 5.3 and 5.7 U/L across treatments, indicating no adverse effects of PJB supplementation on hepatic function. Similarly, TCHO levels varied from 355.0 to 388.7 mg/dL, GLU levels ranged between 103.3 and 107.3 mg/dL, and TP levels ranged from 6.0 to 7.2 g/dL, with no significant differences detected.

### 3.4. Antioxidant Enzyme Activities

Plasma antioxidant enzyme activities of juvenile *P. major* after the 8-week feeding trial are presented in Table 6. Plasma SOD activity was significantly enhanced in fish fed the PJB2.5 diet compared with the level in the control group (*p* < 0.05). However, no significant differences were observed between the PJB5 and PJB10 diets and the PJB0 diet. Plasma CAT levels in PJB2.5 and PJB5 were significantly elevated relative to that in PJB0. Moreover, regarding GSH concentrations, fish fed the PJB2.5 diet exhibited significantly higher values than the PJB0 group. No significant differences were detected among the PJB5, PJB10, and PJB0 diets.

### 3.5. Immune Parameters

The serum lysozyme activity of juvenile *P. major* fed diets containing different levels of PJB for 8 weeks is presented in Table 7. Fish fed the PJB2.5 diet exhibited the highest serum lysozyme activity, which was significantly greater (*p* < 0.05) than those of the control and PJB10 groups. Serum IgM concentrations were not significantly different among dietary treatments (*p* > 0.05). In contrast, serum IL-1 levels were significantly influenced by dietary PJB inclusion (*p* < 0.05). Fish fed the PJB2.5 diet exhibited significantly higher IL-1 levels than the control group (*p* < 0.05), whereas IL-1 concentrations in the PJB5 and PJB10 groups were intermediate and did not differ significantly from those in either the PJB0 or the PJB2.5 group (*p* > 0.05).

### 3.6. Challenge Test

**Figure 1:**
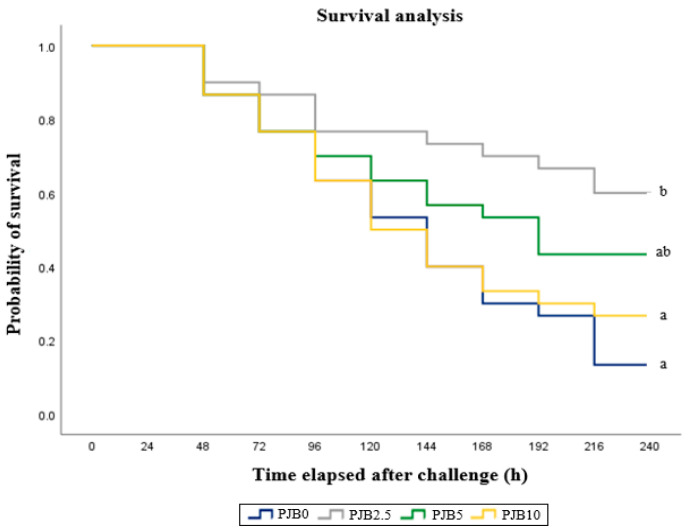
Survival of juvenile red seabream fed experimental diets with different levels of PJB for 8 weeks, and then artificially infected with *Edwardsiella tarda* (means of triplicate experiments ± SE) (*p* < 0.001 for log-rank and Wilcoxon tests). Different letters on the curves indicate significant differences among dietary treatments based on pairwise comparisons (*p* < 0.05).

Pairwise comparisons of survival curves using the log-rank (Mantel–Cox) test revealed significant differences among dietary treatments (Figure 1). Fish fed the PJB2.5 diet exhibited significantly higher survival than those fed the PJB0 and PJB10 diets (*p* < 0.001).

## 4. Discussion

In the present study, dietary supplementation with PJB significantly affected the growth performance of juvenile *P. major*, with clear differences depending on the rate at which PJB was incorporated into the diet. Fish fed the diet containing 2.5 g/kg PJB exhibited significantly higher final weight, WG, and SGR than those in the control group and those with higher PJB rates, indicating that low-level supplementation is effective for promoting growth during the juvenile stage. The growth-enhancing effect observed at 2.5 g/kg PJB is consistent with previous reports on the provision of pomegranate-derived byproducts to aquaculture species. Specifically, improved growth performance following dietary supplementation with pomegranate peel or byproduct materials has been reported in Nile tilapia (*Oreochromis niloticus*), common carp (*Cyprinus carpio*), grass carp (*Ctenopharyngodon idella*), and golden pompano (*Trachinotus ovatus*), particularly when these materials were included in the diet at low to moderate levels [14,15,24,25]. These studies, together with the present findings, suggest that pomegranate byproducts have potential as components of functional feed capable of enhancing growth performance across a range of cultured fish species. Notably, the improvement in growth performance was not accompanied by significant changes in FI, FE, or PER, indicating that the growth-promoting effect of PJB was not driven by increased feed utilization. Comparable responses have been observed in other studies evaluating phytogenic or fruit-derived feed additives, where growth improvement occurred without significant alterations in FI or feed utilization indices [26,27]. These findings suggest that growth improvement may be driven by enhanced physiological functions, including improved digestive capacity and metabolic efficiency, rather than changes in feeding behavior. In contrast, increasing the level of PJB in the diet beyond 2.5 g/kg did not result in additional growth benefits. Fish fed the PJB5 and PJB10 diets showed growth performance comparable to or lower than that of the control group. Similar dose-dependent responses have been frequently reported in studies using plant- or fruit-based feed additives, where excessive inclusion levels failed to enhance growth and, in some cases, negatively affected performance [26,28,29]. These results highlight the importance of optimizing the levels at which agro-industrial byproducts are added to aquafeed in order to achieve growth benefits.

In the present study, dietary supplementation with PJB significantly influenced intestinal digestive enzyme activities in juvenile *P. major*. Among the experimental diets, fish fed the PJB2.5 diet exhibited significantly higher trypsin and lipase activities than those in the control group, whereas amylase activity was not affected by dietary treatment. These results indicate that moderate inclusion of PJB selectively enhanced protein and lipid digestion without altering carbohydrate digestion. The increase in trypsin activity observed in the PJB2.5 group suggests an improved capacity for the hydrolysis of dietary protein. Trypsin plays a central role in protein digestion in marine carnivorous fish, and its activity is closely associated with amino acid availability and growth potential in teleost fish [30,31]. Similarly, the elevated lipase activity indicates enhanced lipid digestion, which is particularly important for marine species such as red seabream that rely heavily on dietary lipids as a source of energy [32]. Enhanced lipid digestion may contribute to improved energy utilization and metabolic efficiency, even in the absence of changes in FI or FE indices. The stimulatory effects of PJB on digestive enzyme activity may be attributable to the presence of bioactive compounds, such as polyphenolic compounds, flavonoids, and organic acids. These compounds have been reported to modulate gastrointestinal physiology by stimulating pancreatic secretion, enhancing bile flow, and improving intestinal function [33,34]. In aquaculture, the inclusion of fruit-derived byproducts rich in bioactive compounds in the diet has been shown to increase digestive enzyme activities in various fish species, including Nile tilapia and grass carp, thereby improving digestive capacity [15,26]. The present findings are consistent with these reports and suggest that PJB can act as a digestive stimulant when incorporated into the diet at an appropriate level. In contrast, the inclusion of higher levels of PJB (5.0 and 10.0 g/kg) in the diet did not result in further enhancement of digestive enzyme activities. This lack of a response at higher PJB levels may be associated with the excessive intake of polyphenolic compounds, which have been reported to inhibit digestive enzymes or lead to the formation of complexes with dietary proteins when they are present at high concentrations [35,36,37]. Such interactions may counteract the stimulatory effects observed at lower inclusion levels, resulting in a plateau or reduction in enzyme activity.

In the present study, dietary supplementation with PJB did not result in significant changes in the whole-body proximate composition of juvenile *P. major*. Moisture, crude protein, crude lipid, and ash contents were comparable among all dietary treatments, indicating that the inclusion of PJB at levels up to 10.0 g/kg did not alter overall nutrient deposition patterns. The absence of significant differences in whole-body composition despite improved growth performance in the PJB2.5 group suggests that dietary PJB did not induce abnormal nutrient accumulation or metabolic imbalance. Instead, growth enhancement appears to have occurred through proportional somatic growth rather than through excessive lipid deposition or altered protein retention. Similar findings have been reported in studies evaluating functional plant-derived feed additives, where an improvement in growth was observed without corresponding changes in whole-body composition [38,39,40]. Such responses are generally interpreted as indicative of balanced nutrient utilization and stable metabolic regulation. Whole-body composition is a sensitive indicator of the long-term nutritional and metabolic effects of dietary manipulation in fish. For example, significant alterations in lipid or protein content are often associated with dietary stress, metabolic disruption, or imbalanced energy allocation [41]. In this context, the stable proximate composition observed across all dietary treatments suggests that PJB supplementation did not adversely affect nutrient partitioning or physiological homeostasis.

In the present study, dietary supplementation with PJB did not significantly affect plasma biochemical parameters of juvenile *P. major*. Plasma levels of AST, ALT, TCHO, GLU, and TP remained comparable among all dietary treatments, indicating that the inclusion of PJB at levels up to 10.0 g/kg did not induce adverse physiological or metabolic effects. Plasma AST and ALT activities are commonly used as indicators of hepatic integrity and cellular damage in fish [42]. Elevated levels of these enzymes are often associated with liver stress or tissue damage caused by nutritional imbalance, toxic compounds, or environmental stressors [42,43]. The absence of significant differences in AST and ALT activities among dietary treatments in the present study suggests that PJB supplementation did not impair liver function or induce hepatocellular damage, even at the highest inclusion level tested. Similarly, plasma TCHO and GLU concentrations were not significantly affected by the inclusion of PJB in the diet. Cholesterol and GLU levels are closely related to lipid and carbohydrate metabolism, respectively, and are sensitive indicators of nutritional and metabolic status in fish [44,45]. The stability of these parameters across treatments indicated that dietary PJB did not disrupt energy metabolism or induce metabolic stress. Total plasma protein reflects the overall nutritional condition and health status of fish, the capacity for synthesizing protein, and the immune status [46,47]. In the present study, the lack of significant variation in TP among dietary treatments further supports the conclusion that PJB supplementation did not negatively affect protein metabolism or physiological condition.

However, in the present study, dietary supplementation with PJB significantly influenced the antioxidant status of juvenile *P. major*, with responses varying according to the level of inclusion in the diet. Fish fed the diet containing 2.5 g/kg PJB exhibited significantly higher plasma SOD activity and GSH concentration than those in the control group, while CAT activity was also elevated in fish fed the PJB2.5 and PJB5 diets. These results indicate that the inclusion of moderate levels of PJB in the diet enhances the antioxidant defense system in juvenile red seabream. Antioxidant enzymes such as SOD and CAT constitute the primary lines of enzymatic defense against reactive oxygen species generated during normal metabolic processes and under stressful conditions such as those experienced by fish in aquaculture systems [48]. SOD catalyzes the dismutation of superoxide radicals into hydrogen peroxide, which is subsequently decomposed by CAT, whereas GSH functions as a key nonenzymatic antioxidant involved in cellular redox regulation [49]. The observed elevations in the levels of these antioxidant components suggest that dietary PJB improved the capacity of juvenile *P. major* to maintain redox homeostasis. The improvement in antioxidant enzyme activities observed in the PJB2.5 group is likely associated with the presence of bioactive compounds in pomegranate byproducts, including polyphenolic compounds, flavonoids, and vitamin C. These compounds have been shown to exert strong antioxidant effects either by directly scavenging free radicals or by upregulating endogenous antioxidant defense systems [50,51,52]. Previous studies have reported similar enhancements in antioxidant status following dietary supplementation with pomegranate-derived byproducts in fish and other aquatic organisms, supporting their status as a natural source of antioxidants [14,15,53]. Notably, the enhancement of antioxidant responses was most pronounced when PJB was added to the diet at a level of 2.5 g/kg, whereas higher levels did not result in further increases in SOD or GSH activity. These results suggest a dose-dependent response to increasing levels of PJB supplementation. Specifically, moderate levels of PJB may effectively stimulate the antioxidant defense system, whereas excessive supplementation may limit further antioxidant effects due to physiological adaptation. High concentrations of polyphenolic compounds exert pro-oxidant effects or reduce the expression of antioxidant enzymes under certain conditions, potentially explaining the lack of further enhancement at higher PJB levels [26,28,54]. Notably, the enhancement of antioxidant enzyme activity occurred without concomitant changes in plasma biochemical parameters, indicating that PJB supplementation did not induce oxidative stress or metabolic disturbance. Instead, the elevated antioxidant capacity observed in the present study likely reflects intensification of the protection against oxidative challenges that fish commonly encounter in intensive aquaculture environments.

In the present study, dietary supplementation with PJB also influenced the innate immune responses of juvenile *P. major*, as reflected by changes in serum lysozyme activity and IL-1 levels. Fish fed a diet containing 2.5 g/kg PJB exhibited significantly higher lysozyme activity than the control group, indicating the enhancement of nonspecific immune defense mechanisms. Lysozyme is a key component of the innate immune system in fish, functioning as a first line of defense against bacterial pathogens through the hydrolysis of peptidoglycan in bacterial cell walls, and its enhanced activity has been shown to be widely associated with improved immune status and disease resistance [2,17]. The significant increase in lysozyme activity observed in the PJB2.5 group suggests that moderate dietary inclusion of PJB can stimulate innate immune function in juvenile red seabream. In addition to lysozyme activity, serum IL-1 levels were significantly affected by the addition of PJB to the diet. Fish fed the PJB2.5 diet exhibited significantly higher IL-1 concentrations than the control group, while intermediate values were observed in the PJB5 and PJB10 groups. IL-1 is a key proinflammatory cytokine in fish that plays a central role in immune cell activation and the initiation of inflammatory responses during pathogen recognition [55]. Moderate upregulation of IL-1 is generally considered indicative of enhanced immune responsiveness rather than pathological inflammation, particularly when not accompanied by adverse physiological effects [56,57]. The absence of significant changes in IgM levels among the dietary treatments in this study suggests that the addition of PJB to the diet primarily influenced innate immune responses rather than humoral adaptive immunity during the experimental period. Similar patterns have been reported in previous studies evaluating phytogenic feed additives, where innate immune parameters responded more rapidly and sensitively than adaptive immune indicators such as IgM [14,17,26,58]. The immune-enhancing effects observed at the 2.5 g/kg inclusion level may be attributable to the bioactive compounds present in pomegranate byproducts, including polyphenolic compounds and flavonoids, which have been reported to modulate immune-related signaling pathways and enhance macrophage and leukocyte activity [17,59,60]. However, the lack of further enhancement at higher inclusion levels suggests a dose-dependent response, where excessive levels of bioactive compounds may not provide additional immunostimulatory benefits. Furthermore, future studies are needed to clarify the immunomodulatory mechanisms of PJB through the analysis of immune-related gene expression.

The present study demonstrated that dietary supplementation with PJB enhanced the resistance of juvenile *P. major* to *E. tarda* infection. Following experimental bacterial challenge, fish fed the PJB2.5 diet exhibited significantly higher cumulative survival compared with the control and PJB10 groups, whereas fish fed the PJB5 diet also showed improved survival relative to the control. These results indicate that dietary PJB supplementation at appropriate levels can effectively enhance disease resistance in juvenile red seabream. *E. tarda* is a major bacterial pathogen in marine aquaculture and causes severe mortality in juvenile *P. major*, particularly under intensive farming conditions [18,61]. Improved survival following bacterial challenge is therefore a strong indicator of enhanced host defense capacity [2,17]. The significantly higher survival observed in the PJB2.5 group suggests that the moderate inclusion of PJB in the diet increased the ability of fish to cope with pathogenic infection. The enhanced disease resistance observed in the present study is likely associated with the improved innate immune responses induced by PJB supplementation. The increased serum lysozyme activity and elevated IL-1 levels observed in fish fed the PJB2.5 diet indicate enhanced early immune activation, which plays a critical role in limiting bacterial proliferation during the initial stages of infection [17,55]. Innate immune parameters are particularly important in determining resistance to acute bacterial infections, as components of the innate immune system respond more rapidly than those of adaptive immunity such as immunoglobulins. In addition to immune modulation, the enhanced antioxidant capacity observed in PJB-supplemented fish may have contributed to improved survival following *E. tarda* challenge. Bacterial infection commonly induces oxidative stress through increased metabolic activity and inflammatory responses, whereas elevated antioxidant enzyme activities can mitigate oxidative damage and help maintain immune cell function during infection [2,49,52]. Therefore, the improved antioxidant status observed at the optimal level of PJB supplementation in the diet may have played a supportive role in enhancing disease resistance. Notably, higher inclusion levels of PJB did not result in further improvement in survival following bacterial challenge. Fish fed the PJB10 diet exhibited lower survival than the PJB2.5 group, suggesting that excessive inclusion of bioactive compounds may not provide additional protective effects. Similar dose-dependent responses have been reported in studies evaluating plant- and fruit-derived immunostimulants, where moderate supplementation enhanced disease resistance, whereas higher levels resulted in diminished or inconsistent effects [14,26]. This highlights the importance of optimizing dietary inclusion levels to achieve maximal immunoprotective benefits.

## 5. Conclusions

The present study demonstrates that PJB can be effectively used as a functional dietary additive for juvenile *P. major* when included at an appropriate level. Here, dietary supplementation with 2.5 g/kg PJB significantly enhanced growth performance, digestive enzyme activity, antioxidant capacity, innate immune responses, and resistance to *E. tarda* infection, without inducing adverse effects on feed utilization, whole-body composition, or plasma biochemical parameters. However, further studies are required to clarify the underlying mechanisms, evaluate the long-term effects of dietary supplementation, and validate the optimal inclusion level under practical aquaculture conditions. Overall, this study suggests the potential of PJB as a sustainable and eco-friendly functional feed additive derived from fruit-processing by-products.

## Figures and Tables

**Table 1:** Vitamin C, total phenolics, total flavonoids, and radical scavenging activities of PJB.

PJB Composition								
Chemical compounds	Vitamin C (mg 100 g^−1^)		10.21 ± 2.240					
	Total phenolics (gallic acid mg 100 g^−1^)		16.15 ± 4.881					
	Total flavonoids (quercetin mg g^−1^)		14.45 ± 6.794					
Radical scavenging activities	Concentration (µg mL^−1^)	4000	2000	1000	500	250	125	IC_50_
	DPPH (%)	76.48	46.81	31.77	24.03	16.94	15.78	4.8
	ABTS (%)	68.50	41.61	23.32	12.67	8.23	3.60	5.0

Abbreviations: DPPH, 1,1–diphenyl–2–picrylhydrazyl; ABTS, 2,2′-azinobis-(3-ethylbenzothiazoline-6-sulfonic acid).

**Table 2:** Composition and proximate analysis of the experimental diets with different levels of PJB (on a dry matter basis).

Ingredients (g/kg)	Experimental Diets			
	PJB0	PJB2.5	PJB5	PJB10
Sardine meal	600	600	600	600
Dehulled soybean meal	100	100	100	100
Wheat flour	205	202.5	200	195
Pomegranate juice byproducts	0	2.5	5	10
Fish oil	35	35	35	35
Soybean oil	35	35	35	35
Vitamin premix ^a^	10	10	10	10
Mineral premix ^b^	10	10	10	10
Choline	5	5	5	5
Proximate composition (%, dry matter basis)				
Dry matter	9.70	9.67	9.72	9.75
Crude protein	5.00	5.01	5.00	4.96
Crude lipid	1.16	1.18	1.18	1.20
Ash	0.97	1.01	1.03	1.02

^a^ Vitamin premix contained the following amount, which was diluted in cellulose (g kg^−1^ mix): L-ascorbic acid, 121.2; DL-α-tocopheryl acetate, 18.8; thiamin hydrochloride, 2.7; riboflavin, 9.1; pyridoxine hydrochloride, 1.8; niacin, 36.4; Ca-D-pantothenate, 12.7; myo-inositol, 181.8; D-biotin, 0.27; folic acid, 0.68; p-aminobenzoic acid, 18.2; menadione, 1.8; retinyl acetate, 0.73; cholecalciferol, 0.003; cyanocobalamin, 0.003. ^b^ Mineral premix contained the following ingredients (g kg^−1^ mix): MgSO_4_·7H_2_O, 80.0; NaH_2_PO_4_·2H_2_O, 370.0; KCl, 130.0; ferric citrate, 40.0; ZnSO_4_·7H_2_O, 20.0; Ca-lactate, 356.5; CuCl, 0.2; AlCl_3_·6H_2_O, 0.15; KI, 0.15; Na_2_Se_2_O_3_, 0.01; MnSO_4_·H_2_O, 2.0; CoCl_2_·6H_2_O, 1.0.

**Table 3:** Growth performance and feed utilization of juvenile red seabream fed experimental diets with different levels of PJB for 8 weeks.

Parameters	Experimental Diets				*p* Value
	PJB0	PJB2.5	PJB5	PJB10	
Initial weight (g fish^−1^)	7.0 ± 0.00	7.0 ± 0.01	7.0 ± 0.00	7.0 ± 0.01	-
Final weight (g fish^−1^)	39.5 ± 0.46 ^a^	41.4 ± 0.12 ^b^	37.8 ± 1.07 ^a^	36.9 ± 0.64 ^a^	0.006
SR (%)	95.2 ± 1.90	96.2 ± 1.90	95.2 ± 1.90	95.2 ± 0.95	0.967
WG (g fish^−1^)	32.6 ± 0.46 ^a^	34.4 ± 0.12 ^b^	30.8 ± 1.07 ^a^	30.0 ± 0.63 ^a^	0.007
SGR (%)	3.69 ± 0.025 ^ab^	3.79 ± 0.006 ^b^	3.59 ± 0.060 ^a^	3.55 ± 0.035 ^a^	0.007
FI	35.1 ± 0.49	37.0 ± 1.41	33.6 ± 1.50	34.7 ± 0.81	0.255
FE	0.92 ± 0.008	0.922 ± 0.034	0.92 ± 0.006	0.87 ± 0.023	0.300
PER	1.72 ± 0.016	1.86 ± 0.074	1.84 ± 0.019	1.63 ± 0.058	0.195
CF	1.95 ± 0.064	1.97 ± 0.067	1.96 ± 0.020	1.93 ± 0.075	0.962
VSI (%)	1.73 ± 0.087	1.80 ± 0.052	1.84 ± 0.074	1.89 ± 0.127	0.687
HSI (%)	1.96 ± 0.060	2.00 ± 0.044	2.01 ± 0.091	1.89 ± 0.115	0.698

Values with different superscript letters within a row are significantly different (*p* < 0.05), whereas mean values in the same row without any superscript are not significantly different. Abbreviations: SR, survival; WG, weight gain; SGR, specific growth rate; FI, feed intake; FE, feed efficiency; PER, protein efficiency ratio; CF, condition factor; VSI, viscerosomatic index; HSI, hepatosomatic index.

**Table 4:** Digestive enzyme activities (mU/mg protein) of juvenile red seabream fed experimental diets with different levels of PJB for 8 weeks.

Parameters	Experimental Diets				*p* Value
	PJB0	PJB2.5	PJB5	PJB10	
Amylase	40.2 ± 2.30	44.0 ± 1.61	39.5 ± 1.57	38.7 ± 3.48	0.445
Trypsin	7.7 ± 0.32 ^a^	14.9 ± 2.51 ^b^	9.8 ± 0.30 ^ab^	9.2 ± 0.82 ^ab^	0.026
Lipase	88.1 ± 8.21 ^a^	131.1 ± 5.36 ^b^	112.1 ± 7.78 ^ab^	102.7 ± 9.93 ^ab^	0.029

Values with different superscript letters within a row are significantly different (*p* < 0.05), whereas mean values in the same row without any superscript are not significantly different.

**Table 5:** Proximate composition (%) and hematological parameters of juvenile red seabream fed experimental diets with different levels of PJB for 8 weeks.

Parameters	Experimental Diets				*p* Value
	PJB0	PJB2.5	PJB5	PJB10	
Moisture	67.6 ± 0.16	67.4 ± 0.25	67.6 ± 0.11	67.6 ± 0.10	0.819
Crude protein	17.4 ± 0.07	18.0 ± 0.19	17.4 ± 0.11	17.4 ± 0.26	0.066
Crude lipid	9.4 ± 0.19	9.3 ± 0.21	9.2 ± 0.11	9.3 ± 0.15	0.853
Ash	4.5 ± 0.04	4.4 ± 0.15	4.5 ± 0.10	4.4 ± 0.09	0.825
AST (U/L)	61.3 ± 3.48	62.0 ± 5.00	63.0 ± 4.04	58.0 ± 6.00	0.887
ALT (U/L)	5.3 ± 0.33	5.3 ± 0.33	5.7 ± 0.33	5.7 ± 0.33	0.802
TCHO (mg/dL)	355.0 ± 15.37	388.7 ± 11.84	372.0 ± 11.59	377.7 ± 15.92	0.429
GLU (mg/dL)	107.3 ± 2.91	103.3 ± 3.18	107.0 ± 4.62	105.0 ± 4.16	0.864
TP (g/dL)	6.4 ± 0.21	6.0 ± 0.52	6.6 ± 0.50	7.2 ± 0.89	0.576

Values are presented without superscript letters, indicating no significant differences within rows (*p* < 0.05), whereas mean values in the same row without any superscript are not significantly different. Abbreviations: AST, aspartate aminotransferase; ALT, alanine aminotransferase; TCHO, total cholesterol; GLU, glucose; TP, total protein.

**Table 6:** Plasma antioxidant enzyme activity of juvenile red seabream fed experimental diets with different levels of pomegranate juice byproducts for 8 weeks.

Parameters	Experimental Diets				*p* Value
	PJB0	PJB2.5	PJB5	PJB10	
SOD (U/mL)	0.54 ± 0.016 ^a^	0.63 ± 0.022 ^b^	0.58 ± 0.018 ^ab^	0.56 ± 0.007 ^ab^	0.019
CAT (nmol/min/mL)	353.5 ± 11.43 ^a^	429.8 ± 6.72 ^b^	427.3 ± 0.84 ^b^	392.8 ± 14.40 ^ab^	0.002
GSH (µM)	5.15 ± 0.030 ^a^	5.51 ± 0.114 ^b^	5.36 ± 0.037 ^ab^	5.33 ± 0.033 ^ab^	0.027

Values with different superscript letters within a row are significantly different (*p* < 0.05), whereas mean values in the same row without any superscript are not significantly different. Abbreviations: SOD, superoxide dismutase; CAT, catalase; GSH, glutathione.

**Table 7:** Immune parameters of juvenile red seabream fed experimental diets with different levels of pomegranate juice byproducts (PJB) for 8 weeks.

Parameters	Experimental Diets				*p* Value
	PJB0	PJB2.5	PJB5	PJB10	
Lysozyme activity (U/mL)	0.073 ± 0.0036 ^a^	0.166 ± 0.0188 ^b^	0.114 ± 0.0060 ^ab^	0.090 ± 0.0123 ^a^	0.003
IgM (mg/mL)	2.9 ± 0.22	3.1 ± 0.23	3.2 ± 0.20	3.1 ± 0.16	0.714
IL-1 (pg/mL)	235.3 ± 5.27 ^a^	261.2 ± 7.13 ^b^	251.7 ± 5.27 ^ab^	239.9 ± 3.97 ^ab^	0.040

Values with different superscript letters within a row are significantly different (*p* < 0.05), whereas mean values in the same row without any superscript are not significantly different. Abbreviations: immunoglobulin M; IL-1, interleukin-1.

## Data Availability

The raw data supporting the conclusions of this article will be made available by the authors on request.
